# Effect of Vigorous Physical Activity on Executive Control in Middle-School Students

**DOI:** 10.3390/ijerph16203949

**Published:** 2019-10-17

**Authors:** David S. Phillips, James C. Hannon, Bradley B. Gregory, Ryan D. Burns

**Affiliations:** 1 Secondary and Physical Education Department, Salisbury University, Salisbury, MD 21801, USA; dsphillips@salisbury.edu; 2 College of Education, Health and Human Services, Kent State University, Kent, OH 44242, USA; jhannon5@kent.edu; 3 Department of Psychology, Palm Beach Atlantic University, West Palm Beach, FL 33401, USA; brad_gregory@pba.edu; 4 Department of Health, Kinesiology, and Recreation, University of Utah, Salt Lake City, UT 84112, USA

**Keywords:** cognitive control, intervention, physical activity, schools, students

## Abstract

The purpose of this pilot study was to examine the acute effect of vigorous physical activity on executive control in eighth grade students from the U.S. Participants were eighth grade students (*N* = 68; 26 girls, 42 boys) recruited from one middle school located in the Mountain West region of the U.S. Two groups of participants were assigned to receive either a vigorous physical activity or a sedentary condition within a counter-balanced cross-over design using a 2-week washout. Both groups were administered Trails Making Tests A (TMT-A) and B (TMT-B) at 20- and 25-min post-treatment, respectively. Mixed design ANOVA tests with repeated measures examined differences between treatments on TMT-A and TMT-B performance and the modifying effect of sex. Students who completed the physical activity condition displayed a faster time to completion on the TMT-B compared to students who completed the sedentary condition (Mean difference = −6.5 s, *p* = 0.026, d = 0.42). There were no differences between treatment groups on TMT-A and no sex × treatment interactions (*p* > 0.05). This pilot study suggests that vigorous physical activity may improve executive control in middle-school students and adds to the existent literature that continues to examine the emerging link between physical activity and cognition in school-based settings.

## 1. Introduction

Core cognitive processes collectively termed ‘Executive Control’ include inhibition, working memory, and cognitive flexibility—functions mediated by pre-frontal cortex networks that ‘control’ non-routine behaviors [1]. These executive control functions are integral elements for learning [2]. Within the child-aged controlled laboratory setting literature, researchers have examined the relationship between physical activity (PA) and executive control using a number of cognitive instruments to measure both the speed and accuracy of response, with PA as a stimulant for learning [3]. Studies have reported a wide array of cognitive improvements after PA including facilitating information processing, decreasing response times [3,4], and influencing executive control performance, but not in the predicted U-shape response function [5].

Within the Physical Education (PE) literature, cross-sectional correlational approaches have historically been utilized to determine plausible associations among PA, executive control, and youth academic achievement. In many seminal chronic-PA based studies, test results have been correlated with Grade Point Average and health-related fitness testing scores, particularly the fitness domain of cardiorespiratory endurance [6,7], across student populations encompassing large sample sizes. There have also been a number of translational studies that have considered an acute causal relationship between PA and improvements in executive control within an authentic real-world setting, the most recent using more academic-based tests (such as math tests) as the assessment instrument [8]. However, only a small number of studies have considered how PA may affect executive control in school-settings using scientifically validated assessment instruments. Previous studies in lab settings have involved participants completing doses of exercises on a treadmill or stationary bike [9,10]. Intensities using treadmills and exercise bikes may not reflect the sporadic and transient nature of pediatric physical activity in naturalistic settings. Additionally, lab assessments have included the use of electroencephalograms or magnetic resonance imaging to measure neural activation [11,12]. Lab exercises and assessments may have weak ecological validity when generalizing results to school settings; therefore, more real-world applied research is needed in this growing field. Previous studies have tended to focus on the ‘dose-response’ between PA and executive control, ranging from reported high doses of PA (>40 min) leading to higher executive control post-test scores [13], and a PA intensity above the vigorous target heart rate zone yielding beneficial effects on executive control performance [14]. These past results suggest that the optimal PA dosage needed for an increase in executive control may be close to an individual’s anaerobic threshold.

Despite the aforementioned findings, there are limited studies that have reported an improvement in the Trails Making Test (TMT) within the pediatric population. Again, in terms of intensity, it has been suggested that TMT improvement is associated with children who spent time above their aerobic target heart rate zone during an after-school ‘Fitness Improves Thinking’ intervention [15]. However, despite positive findings in the current research, no study has focused on TMT performance using a PA stimulus during middle-school PE class. The current study could be an important contribution in validating both PA opportunities and the importance of PE within the educational curriculum. Therefore, the primary purpose of this pilot study was to examine the effect of an acute bout of vigorous intensity PA on TMT-A and B performance in middle-school students from the U.S. It was hypothesized that TMT-A and B performance will increase following a single bout of vigorous PA during PE and remain unchanged or decrease following a period of sedentary behavior. 

## 2. Materials and Methods 

### 2.1. Participants

Participants were a convenience sample of students enrolled in eighth-grade PE classes from a middle school located in the Mountain West region of the U.S. Based on the results from an a priori power analysis using a medium-sized effect and an alpha level set at *p* < 0.05, a sample size of 34 participants per treatment group was needed to achieve at least 80% statistical power. After participant dropout (absences/injury) the number of participants with complete data in the current study was *N* = 72, with *n* = 36 within each treatment group: group 1 (*n* = 36; males = 21, females = 15) and group 2, (*n* = 36; males = 23, females = 13). Using a counter-balanced cross-over design, in-tact classes were assigned to a treatment of vigorous PA during PE or a sedentary condition consisting of sitting and watching a video related to PE content. Participants who were unable to participate in PE lessons without modifications were excluded from this pilot study. Group 1 performed the PA condition first and the sedentary condition second, while group 2 performed the sedentary condition first and PA condition second. Permission to conduct the pilot study was obtained from the University Institutional Review Board, the school district, the school administration, and teachers prior to the start of data collection. The participants provided written informed assent and parents provided written informed consent prior to data collection. 

### 2.2. Instrumentation

The Trail Making Test A (TMT-A) and Trail Making Test B (TMT-B) are two tests based on the Army Individual Battery Test from 1944. Historically, the TMT-A and B have predominantly been used as a measurement instrument for neuropsychological research to distinguish brain-damaged patients from healthy control participants. The TMT-A (congruent task) and TMT-B (an incongruent task where the participant has to elicit inhibitory control due to interference) include connecting 25 numbers (in a dot-to-dot process) spread out on an A4 piece of paper, in numerical order, as quickly as possible. Test B (incongruent task) is exactly the same as Test A (congruent task) except for the inclusion of letters; the participant must alternate between consecutive numbers and letters in order to complete the task. Test-B is 56.9 cm longer than Test A; therefore, it takes longer to complete, and there are at least one or more items that are located in the pathway of the trail. 

TMT-A and TMT-B have often been used measurement tools in neuropsychological research [16]. Yet the construct validity of the instrument has been questioned due to the complex nature of the cognitive mechanisms underlying TMT-B and lack of consensus of what the test actually measures. Yet the components of executive control that the TMTs are purported to measure have always been consistent, but researchers have chosen to focus on certain measurement elements in the last 30 years, from visual perception and fine motor abilities [17], visual/nonverbal intelligence and spatial abilities [18], visual attention and processing speed [19], to task set inhibition [20]. The TMT-A is a low-functioning cognitive test that requires visuo-perceptual capabilities [21] whereas the TMT-B is a high-functioning cognitive test that adds higher level cognitive control skills [22] that of mainly working memory, and then task switching (consecutively switching between numbers and letters). 

Due to the instrument’s application within brain health research most studies have involved the senior population as the target group, concentrating on aging executive control decline. Other results suggest that poor performance of TMT-B is a predictor of walking speed in older adults [23].

There are four different forms of the TMT. The Intermediate form (youth specific) of the TMT-A and B was chosen, from the Halstead-Reitan neuropsychological test battery [24] to align with the age of the participants. The benefits of using the TMT-A/B as the measurement instrument are numerous; the tests can be feasibly used in a school setting; they are short in duration and are cost effective; the protocols of test-retest are easy to administer, and children are familiar with dot-to-dot matrices. 

In this study, the researcher compared the time (in seconds) for each individual test taken within the two different treatments. The TMT test-retest reliability was shown to be at Intraclass Correlation Coefficient (ICC) = 0.79 for Test A, and Intraclass Correlation Coefficient ICC = 0.89 for Test B [24]. E600 Polar Heart Rate monitors were used to assess PA intensity during the PA condition and were pre-programmed at a calculated 70–85% age predicted maximum heart rate as per Centers for Disease Control (CDC) guidelines [25]. Each participant was instructed to maintain their heart rate within the target zone (144 bpm to 175 bpm) while working physically at each station of the aerobic circuit for one minute. Heart rate monitors were not worn during the sedentary condition. Research has suggested that Polar Heart Rate Monitors yield energy expenditure estimates more accurately than in other calibrated devices with high test-retest reliability (ICC = 0.86 to 0.99) in school-based research [26,27]. 

### 2.3. Procedures

Two initial visits were made to the research site prior to data collection. The first visit was to acclimate the participants to wearing heart rate monitors during their PE lessons. During the second visit, the participants were familiarized with the aerobic circuit that would be used as the PA condition. This was done to enhance fidelity of the treatment. Students were also shown methods to increase or decrease heart rate in order to stay within the target heart rate zone. The PA condition included the participants wearing the heart rate monitors and participating in twenty minutes of vigorous intensity PA via an aerobics circuit. The intensity was set at 70–85% of age-predicted maximum heart rate. This intensity was preprogrammed on each heart rate monitor for each participant. The monitor was set to beep if a respective participant did not maintain the designated heart rate within the exercise session. In order to yield vigorous PA within the PE lesson, an aerobics circuit was designed based on the previous fitness literature [28]. Aerobic circuits used were incorporated into normal PE lessons at the research site; therefore, the exercises were familiar to the participants. The aerobic circuit included nine aerobic stations that were spaced within a gymnasium (see Table 1). The stations were organized to concurrently accommodate four participants, working for a one-minute period, with a trained assigned research assistant at each station supervising each activity. The participants rotated to the next station within a 7 s transfer time. 

Although the participants were ‘switching tasks’ during the aerobics circuit, there is no reported evidence within the literature to suggest that this would have an advantageous effect on the task-switching nature of the TMT-B. A mediator that seems to be a component of the PA-executive control relationship is elevating the heart rate within the vigorous heart rate zone [13,14]. Elevated heart rate indicates a higher exercise intensity, which may manifest additional multi-level physiological mechanisms to improve executive control including increasing in brain-derived neurotropic factor (BDNF) and insulin-like growth factor-1 (IGF-1) at the molecular level, angiogenesis and neurogenesis at the cellular level, and improvements in gray matter volume and hippocampal volume on the structural level [29]. The concept of switching from one aerobic task to another within the circuit was to stave off volitional fatigue by overworking one particular muscle group, and alleviate boredom from one single aerobic task, a common practice in PE class.

At the end of the 20 min aerobic circuit, the PA group returned their heart rate monitors to the researcher so the data could be downloaded confirming that the participants remained within the target heart rate training zone. The participants then dressed out in the locker room, and walked to a classroom, imitating normal practice after a PE class. The PA condition group then sat in silence, with no communication allowed between peers, the researcher, and research assistants, and TMT-A was completed at 20 min post treatment. They then sat quietly, reading, and then had the TMT-B administered at 25 min post treatment. At completion of the test, the participants were dismissed and returned to class. Research assistants timed the tests for each participant individually.

The sedentary treatment included the group assigned to the non-active condition being taken, by the researcher, to a classroom. During the treatment, the participants watched a video in silence for 20 min, with no communication with the researchers, teachers, or peers allowed. This condition was employed to maintain heart rate at approximately resting levels. Immediately after the non-active condition (i.e., after a total of 20 min sedentary behavior) participants completed TMT-A and then sat quietly reading, and then had the process repeated with TMT-B administered at 5 min post non-active condition (i.e., after a total of 25 min sedentary behavior). Graduate assistants timed the tests for each participant individually. At completion of the test the participants were dismissed and returned to class. Approximately two weeks were provided (a washout period) before testing conditions alternated for group 1 and group 2. 

Testing was conducted in the manner of a regularly scheduled PE class in whereby some students were sitting in class and took the test, while some students were in the gym being physically active. This authenticity was compounded and repeated during the second visit (after a passage of two weeks of time to prevent learning effects) where the researcher collected data using the same protocol as the first visit, except with the two classes switching treatments. 

### 2.4. Statistical Analysis

Data were screened for outliers using z-scores and box plots and checked for Gaussian distributions using k-density plots. Approximately four participants with complete data were dropped because of outlier scores (5.6% of the sample). Therefore, the final sample size was *N* = 68, with *n* = 34 within each treatment group. Two separate 2 × 2 × 2 mixed design Analysis of Variance (ANOVA) tests with repeated measures were used to examine the effect of treatment (PA, sedentary) on TMT-A and TMT-B performance. Carry-over effects were examined using an order term within the factorial design (PA condition first, sedentary condition first). The possible modifying effect of sex (female, male) was also tested using a sex × treatment interaction term. Sex and order were between-subjects’ factors and treatment was the within-subjects factor. Effect sizes were computed using Cohen’s d with <0.2 representing a small effect, d ≈ 0.5 representing a medium effect, and ≥ 0.8 representing a large effect. Statistical significance was set at *p* < 0.05 for all statistical tests. Analyses were conducted using SPSS v24.0 statistical software package (IBM, Armonk, NY, USA).

## 3. Results

All participants in the PA condition were within their target heart rate zone for the entirety of the treatment (100% adherence). Average heart rate during the PA condition was 157.5 bpm. The descriptive data are presented in Table 2 and the results from the mixed-design ANOVA test are presented in Table 3. For TMT-A testing performance, there were no statistically significant differences between treatment groups (F = 1.5, *p* = 0.226) and no statistically significant sex × treatment interaction (F = 2.1, *p* = 0.155) or order main effects (F = 0.2, *p* = 0.631). However, for TMT-B testing performance, there was a statistically significant treatment main effect (F = 5.1, *p* = 0.026). Students who completed the PA condition displayed faster response times (i.e., faster time to completion) on the TMT-B compared to students who completed the sedentary condition (Mean difference = −6.5 s, *p* < 0.05). This difference approached a medium-sized effect (Cohen’s *d* = 0.42). There was no sex × treatment interaction (F = 1.9, *p* = 0.170) and no order effect (F = 0.1, *p* = 0.770) within the ANOVA model for TMT B performance.

## 4. Discussion

The purpose of this pilot study was to examine the effects of an acute bout of vigorous intensity PA on TMT-A and B performance in U.S. middle-school students. We hypothesized that performance on both the TMT-A and B tasks would improve (i.e., a faster time-to-completion) following a single bout of vigorous PA during PE and remain unchanged or possibly decrease following sedentary behavior. Results were that TMT-B performance improved after the vigorous PA condition relative to the sedentary condition. Findings from this pilot study support the current literature that suggests that brief bouts of vigorous PA during the school day can be beneficial for improving cognitive functioning in school-settings. This novel study is an attempt in examining the association between PA and executive control in an authentic environment, using a scientifically rigorous cross-over experimental design. 

Although the data reported statistical significance for the effect of an acute bout of vigorous PA on TMT-B performance, results should be considered from a cautionary perspective. The trigger mechanism that may explain how PA affects executive control is partially unknown; however, evidence has shown that habitual and acute PA and exercise may improve attention span and working memory by altering the neurochemicals serotonin, dopamine, norepinephrine, in addition to brain-derived neurotrophic factor, synaptic proteins, and insulin-like growth factor-1 [30,31]. Indeed, the acute effect of PA on cognitive functioning also seems to be dose-dependent, in that PA or exercise of higher intensities have a more significant beneficial impact on tests of cognitive function than PA or exercise of lower intensities [32,33]. The duration, intensity, and time frame for improved executive control performance, known as the dose-response, is individualistic by nature, due to fitness levels, social-economic status, BMI, adiposity, and trajectory of cognitive development [34,35,36,37]. It is also logically valid to suggest that within the primary endeavor of learning, children are more attuned to academic testing, rather than testing executive control skills within the academic classroom. 

There are some interesting questions that arise from the results of the current study. It is becoming clear in the literature that children are not affected in the same U-shape PA-induced arousal pattern as adults, as bouts of high intensity PA do not interfere with learning in a negative manner [3]. Previous research suggests that lasting effects on cognition may occur anytime between 16 to 48 min post PA condition [13,38], leading to questions regarding an ‘optimal time’ for improved executive control (and the positive results from the current study fall within this theoretical construct). It has also been widely reported that TMT-A is a low-functioning cognitive test that is not a predictor of overall cognitive abilities and measures differing cognitive demands as opposed to TMT-B. Therefore, a lack of statistical significance using the TMT-A should be expected [5]. Some researchers have reported that a component of executive control that the TMT-B measures, task-switching, is not been as sympathetic to change, after a bout of PA, as compared to other instruments such as the Stroop Test [39]. However, in the current study, the average time to completion of the TMT-B after a bout of vigorous PA was 6.5 s faster than after the sedentary condition, aligning with current PA-TMT research [15,40,41]. These differences did approach a medium-sized effect and thus may be practically meaningful.

The strengths of this pilot study include the targeting of a vigorous PA intensity assessed using objective heart rate monitoring. Another strength was the use of an experimental cross-over research design and improves internal validity evidence that the PA condition directly effects performance on the TMT-B. The intervention was also administered within a real-world setting, which provides strong ecological validity evidence for the results. However, there are limitations to this pilot study that must be considered before any generalizations can be made. First, this sample comprised of eighth grade students located from the Mountain West region of the U.S.; therefore, the external validity of the results is questionable if generalized to younger or older age groups and students from different regions. Second, although the sample yielded adequate statistical power, having a larger sample size may have manifested results with greater internal validity that is needed to establish efficacy. Third, within cross-over research designs there is always a possibility of a carry-over effect between study periods. Although the order effect from the ANOVA was not statistically significant, there may have been learned behavior between study periods, such as TMT test familiarity, which may have confounded the results. Fourth, the nature of school-based design increases limitations related to scientific rigor; however, the lack of previous child-based research in authentic settings hinders the translation debate [42]. Also related to the research design, baseline or pre-test data were not collected during each study phase, precluding the analysis of change which is recommended when employing future non-pilot efficacy studies. Furthermore, use of the age-predicted maximum heart rate normally used to calculate exercise intensity provides only an estimate of maximum heart rate; use of measured maximum heart rate may have improve the internal validity of the results. Use of the rate of perceived exertion may have provided an alternative means to estimate exercise intensity. Fifth, the exercise exposure was highly variable in that many different exercises were used, each having its own unique effect on heart rate and possibly other mediators of executive control. A more homogenous exercise exposure may have provided greater specificity of the observed results. Finally, it is now commonly believed that executive control is a complex, multi determined construct, with many separate cognitive skills, that requires different types of measurement tests to measure different types of cognitive processes. Within this paradigm lies a major limitation of this study - admittedly, a stand-alone single cognitive measurement pencil/paper test of convenience such as the TMT-A and B has a limited focus on measuring cognitive processes, and although it is an efficient way to collect data, results are further diminished by the nature of a school-based design, which increases limitations related to scientific rigor. 

## 5. Conclusions

Bouts of vigorous PA may improve cognitive health in school-aged children, and as cognitive performance is essential for learning, physical education classes with aerobic-based PA opportunities may support a learning role within the educational curriculum. PA opportunities throughout the school day should be considered as an integral conduit in not only aiding academic behaviors and performance, but just as importantly, to the development of overall mental health of young people, in a setting where they are present for more than six hours a day (school).

This study was an attempt to translate past laboratory-based findings into an authentic field-based context. Elements of executive control, of selective attention, inhibition, task switching, working memory and manipulation, and understanding context are integral to success in the classroom and are a better predictor of math and reading success than intelligent quotient tests [2,43,44,45]. It is therefore salient to consider the effect of PA on assisting school-aged children with the ability to plan, schedule, and deal with attention/focus interferences within the classroom, and possible consequent effect on academic performance. 

This study should be viewed as a positive step forwards in endeavoring to consider questions that until of late, have been relatively unanswered. In essence, improvement in performance of the TMT-B as a cognitive instrument used with children in a school-based setting after a bout of PA should be considered a marker of improved executive control performance. The current study aligns with recent literature that suggests vigorous PA may trigger mechanisms that lead to better executive control and possibly better academic achievement. This study directly contributes to the growing body of knowledge considering how higher levels of PA as an appropriate vehicle for executive control change in youth, and to the continual striving to translate the laboratory into the classroom to inform educational policy and practice.

## Figures and Tables

**Table 1:** Description of the employed aerobics circuit during the physical activity treatment condition.

Name of Exercise	Description
Line Jumps	The participants will jump sideways across and back over a line with both feet together.
Ladder Run	Running through an agility ladder/set of cones, and then run down the side of the ladder/cones to repeat the process.
Hurdles	The participant will hurdle over small 6-12-inch hurdles and then run down the side of the hurdles to repeat the process.
Step Ups	The participant will step up and down on 18-inch-high aerobic steps.
High Knees	The participant will lift alternate knees high into the air on the spot.
Shuttle Drills	The participant will sprint to cones and back, 3, 5 and 10 yards away.
Z Pattern Run	The participant will run through a set of cones arranged in a zig-zag pattern 5 yards away from each other, and then run down the side of the cones to repeat the process.
Jump Rope	The participant will jump rope on the spot.
Jumping Jacks	The participants will complete jumping jacks on the spot.

**Table 2:** Trail Making Test A and B performance (in seconds) for the total sample, within sex groups, and within allocation group order (means and standard deviations).

Sample	Test Type	TMT-A Post SA		TMT-A Post PA		TMT-B Post SA		TMT-B Post PA	
	N	Group1	Group2	Group1	Group2	Group1	Group2	Group1	Group2
Total Sample	68	23.2 (6.4)	20.8 (3.9)	20.7 (6.7)	24.5 (6.6)	57.5 (18.8)	48.0 (17.0)	46.9 (15.5)	48.7 (10.2)
Male	42	23.8 (7.0)	21.1 (4.0)	20.7 (7.5)	24.1 (4.3)	58.3 (21.7)	50.5 (13.5)	46.9 (17.4)	47.5 (9.3)
Female	26	22.2 (5.4)	20.3 (3.8)	20.8 (5.1)	25.3 (9.0)	55.2 (12.6)	44.5 (20.9)	47.0 (12.0)	50.5 (11.5)

*Note:* SA stands for sedentary activity; PA stands for physical activity; TMT-A stands for Trail Making Test A; TMT-B stands for Trail Making Test B; Group is the group order allocation number.

**Table 3:** Analysis of Variance table for TMT-A and TMT-B test scores.

Test	Effect	F-Statistic	*p*-Value	*Eta-Squared*
TMT-A	Treatment	1.5	0.226	0.005
	Sex	0.6	0.461	0.001
	Sex × Treatment	2.1	0.155	0.006
	Order	0.2	0.631	0.004
TMT-B	**Treatment**	**5.1 ^†^**	**0.026**	**0.018**
	Sex	0.5	0.501	0.002
	Sex × Treatment	1.9	0.170	0.012
	Order	0.1	0.770	0.014

*Note:* TMT-A stands for Trail Making Test A; TMT-B stands for Trail Making Test B; bold and ^†^ indicates statistical significance, *p* < 0.05.
